# Implications of COVID-19 Vaccine Hesitancy: Results of Online Bulletin Board Interviews

**DOI:** 10.3389/fpubh.2021.757283

**Published:** 2022-01-10

**Authors:** Jack M. Gorman, Sara E. Gorman, William Sandy, Nellie Gregorian, David A. Scales

**Affiliations:** ^1^Critica Inc., Bronx, NY, United States; ^2^Fluent LLC, New York City, NY, United States; ^3^Department of Medicine, Weil Cornell Medical College, New York City, NY, United States

**Keywords:** COVID-19, vaccine, online, hesitancy, trust

## Abstract

Reluctance to accept vaccination against COVID-19 poses a significant public health risk and is known to be a multi-determined phenomenon. We conducted online focus groups, or “bulletin boards,” in order to probe the nature of COVID-19 vaccine hesitancy and its implications. Participants were 94 individuals from three distinct U.S. geographical areas and represented a range of demographic and socioeconomic characteristics. Six themes emerged from the 3 day-long bulletin boards: the most trusted source of health information sought is the personal physician; information about health is nevertheless obtained from a wide variety of sources; stories about adverse side effects are especially “sticky”; government health institutions like CDC and FDA are not trusted; most respondents engaged in individualistic reasoning; and there is a wide spectrum of attitudes toward vaccination.

## Introduction

The introduction of safe and effective vaccines that protect against the virus that causes COVID-19 has the potential to bring the pandemic under control. Unfortunately, a substantial minority of Americans are either hesitant to be vaccinated or say they will absolutely not receive one of the vaccines under any circumstances (1). Vaccine hesitancy and refusal threaten the ability to establish community (also called “herd”) immunity and therefore pose a significant risk to the public's health (2).

Vaccine hesitancy and refusal are complex phenomena involving multiple themes and narratives (3–5). These phenomena vary by ethnic and racial groups, geographic areas, political affiliations, and a host of other demographic and cultural factors (6–12). Vaccine hesitancy and refusal have been fueled in part by misinformation and disinformation about COVID-19 that has been spread throughout traditional and social media (13–15). Misinformation about vaccines is particularly difficult to dislodge (15–17).

While survey data have provided important information about the reasons for vaccine hesitancy, direct interviews and focus groups offer the potential to reveal more nuanced factors and to place vaccine hesitancy within a larger socio-ecological context. Among the reasons cited for vaccine hesitancy in one recent focus group study are concerns with the rapid development of the vaccine and fears about long-term adverse side effects (18). The same research group found that Black focus group participants also cited mistrust of the healthcare system and racial injustice as reasons for vaccine hesitancy (18).

In the present work we conducted online focus groups, or bulletin boards, in an attempt to probe more deeply into why people are skeptical about COVID-19 vaccines. Efforts to persuade people to accept vaccination are more successful among those who are hesitant to be vaccinated but not yet fully decided against vaccination (19). One recent study did find that people who were “strongly hesitant” about vaccinations against COVID-19 could be persuaded by messages that “highlighted the personal benefits of vaccinations or directly addressed speed of developmental concerns” (20). We used an initial screener to select participants who were hesitant to receive a COVID-19 vaccine but not yet firmly decided for or against having one. Our analysis of the results was influenced by the social ecological model (21). The information gleaned from these interviews revealed several themes that we believe are important not only to understanding COVID-19 vaccine hesitancy and refusal but also to larger issues facing the U.S. healthcare system.

## Methods

Instead of the originally planned in-person focus groups, which became impossible during the COVID-19 pandemic, we drew upon the extensive literature about online focus groups (22–24), which we call “bulletin boards.” A bulletin board is an asynchronous discussion involving greater numbers of individuals than typical focus groups and taking place over an extended period of time. Participants log into a password-protected site to answer questions that are posted and monitored by a moderator, who can also follow up on responses for clarification or elaboration.

The online bulletin board is a flexible research tool that allows a moderator to post questions and probe any individual participant following their entry. The respondents can take as much time to respond as they need. Individual responses are uninfluenced by the group, as participants do not see other responses to any given question until after they have posted their own response.

We conducted the bulletin boards for this study between January 12 and January 28, 2021. We recruited 94 individuals aged 18 and older from three regions in the U.S.: Newark NJ, Chicago Il, and Central Texas. These areas were chosen to offer geographical diversity. Participants were chosen who specifically expressed uncertainty or hesitancy about having a COVID-19 vaccine.

Participants in the online bulletin board interviews were recruited from a panel of U.S. consumers. Panels consist of individuals who agree to periodically participate in market research studies. To ensure a diverse composition of such panels, that is nationally representative, they are recruited through a wide variety of channels: in-person, via e-mail, through social media, mobile apps, web banners, print media, billboards, telephone, radio, and referrals. Such panels provide a large pool of potential respondents for diverse research needs.

For the purposes of this study on vaccine hesitancy, members of an existing panel of U.S. consumers were screened by criteria specific to the desired sample. Those criteria included attitudes toward vaccines in general, combined with intentions regarding COVID vaccination. In addition, they were screened to meet certain demographic criteria, to ensure diversity including gender, age, ethnicity, income, and level of education.

Individuals meeting all of the desired criteria were then invited to participate in the study, and offered an incentive for doing so. At this point, they could either opt in or opt out. If they opted to participate, they were required to provide written consent. These individuals were then provided the time and date of the interview and a link to use to join a secure online platform where the interviews took place.

Subjects were excluded who had already been vaccinated, were planning to be vaccinated, or had decided they absolutely would not be vaccinated. They were also excluded if they indicated that they considered all vaccines to be unsafe or unnecessary. If they qualified, they were asked to participate in the bulletin board over the course of 3 days, log in at their convenience and answer questions that would be posted by the moderator. Participants were also informed that they were free to end their participation at any time, and free to leave any question unanswered.

Due to the length of the interviews and the need to take part for three consecutive days, participants were offered compensation of $120.00—a standard incentive for bulletin board recruitment in the U.S. Compensation was reasonable based on the inconveniences and time commitment imposed by participation the study. We do not believe it to have been coercive or to have presented undue influence over participants.

This research was deemed exempt from IRB review by Ethical and Independent Review Services and approved by the Weill Cornell Medical College IRB.

During the bulletin boards, we asked participants about their attitudes and behaviors with respect to both the influenza and COVID-19 vaccines, but only results concerning COVID-19 vaccines are reported here. Questions were posed to the participants in order to explore the following:

1. Sources of medical information and advice that help shape opinions
2. Sources that are most trusted and relied upon
3. Attitudes toward public health authorities
4. Behaviors and attitudes toward the COVID-19 vaccine
5. Drivers of these attitudes and of COVID-19 vaccine hesitancy
6. Categories of vaccine hesitancy.

The three bulletin board conversations, with 94 participants across 3 days yielded a wealth of information and several hundred pages of transcripts. Several themes and patterns emerged through analysis of these qualitative data.

Data quality control for this study was implemented in several ways. The study employed purposive sampling, screening to ensure that respondents reflected the target population in terms of demographic, behavioral, and attitudinal characteristics. The interviews were conducted by trained moderators, each with 20+ years of qualitative research experience. Research questions were posted for participants, who were then allowed to respond at their convenience over the course of three consecutive days. This method minimizes time pressure and allows for more thoughtful responses. The responses of other participants were not revealed until after each participant had submitted his/her own responses. This method helps to minimize the influence of participants on one another's responses.

A combination of methods was employed in the analysis of more than 600 pages of transcripts generated by the online interviews, including qualitative content analysis, narrative analysis, and interpretive phenomenological analysis (IPA). These methods enabled us to explore how respondents narrate and make sense of their prior experiences with vaccines, with medical professionals, and with various sources of medical information/influence, and how they rationalize their behaviors and opinions with respect to Covid vaccination. Such analysis also enabled us to identify the range of opinions exhibited, how different perceptions tend to cluster or aggregate, as well as which opinions are universally shared, and which are more idiosyncratic.

## Results

As shown in Table 1, the participant sample (*n* = 94) represented considerable diversity with respect to key demographic variables, including gender, race/ethnicity, marital status, education level, and household income.

**Table 1:** Characteristics of 94 participants in bulletin board discussions.

**Gender**	
Female	54
Male	39
Non-binary	1
**Race/ethnicity**	
Caucasian/White	43
African American/Black	23
Hispanic/Latinx	12
Asian American/Pacific Islander	10
Other (Bi- or Multi-racial)	3
Middle Eastern	1
West Indian	1
Native American	1
**Marital status**	
Single	49
Married	30
Divorced, widowed, or separated	15
Education level
High school graduate or less	9
Some college	40
College graduate	29
Some post graduate	15
**Household income level**	
Under $35,000	21
$35,000–$49,999	28
$50,000–$74,999	19
$75,000–$99,999	17
>$100,000	13

Six themes emerged from this research among these vaccine-hesitant online bulletin board participants.

### Trusted Sources

When actively seeking health information, one's own personal physician was clearly identified as the most trusted source. Also important were other healthcare professionals (e.g., pharmacists, dieticians, and physical therapists). A typical comment was “I trust my physician the most because with regards to my health she's been caring for me since I was born.” An important caveat, however, is that a personal experience, such as a family member having an adverse side effect to a vaccine, is often sufficient to override the primary healthcare provider's advice. Participants also sought out health information from online medical sources, and most frequently cited WebMD.com, Healthline, MayoClinic.org, and the CDC site.

### People Obtain Health Information From a Wide Variety of News Sources of Varying Quality

Participants reported obtaining information from a wide array of news sources. Online news and social media were the top sources of news, while only about one-third of participants cited network or cable news as their main sources. Unmediated news sources, sometimes taking the form of anecdotal or unsubstantiated news posted and shared on social media were reported to be one of the participants' top two sources for news. Such news sources often serve as sources of medical information, including information about the COVID vaccine, even if people are not necessarily using them for this purpose. That is to say, people turn to a limited set of medical sites and medical professionals when actively seeking out medical information but are *exposed to* influential medical information from a far wider set of media sources, whether they are looking for it or not. Mere exposure to misinformation or alarming stories about vaccines was often effective in instilling or maintaining vaccine hesitancy.

### The “Stickiness” of Alarming Stories About Severe Vaccine Reactions

Once exposed to such alarming stories, they become “sticky” and linger in respondents' minds, seeding doubt and concern, and undermining assertions of vaccine safety. Participants felt overwhelmed with the amount of often-contradictory information they have received about COVID-19 from myriad sources. This sense of feeling overwhelmed by so much information often results in confusion and reinforces vaccine hesitancy. They feel they know a lot about the pandemic, and do not perceive a knowledge deficit to be a major factor in COVID vaccine hesitancy. Rather, the “stickiest” information comes from negative stories heard about adverse effects of the vaccines. A single story about a serious adverse reaction often has much greater influence on the decision to be vaccinated than reams of scientific data, or than the equally frightening, and far more numerous, stories of the effects of COVID itself. For example, one participant explained that “the one thing I read was on the news about people having severe allergic reaction to corona vaccine and one doctor in Florida died of this vaccine, which caused a concern for me.” Another said, “I've heard people collapsing after the vaccine.”

### Distrust of Various Medical Institutions

Many expressed distrust of the medical industry and medical institutions, as well as of “Western” medicine. As one participant said, “I question anything from any western doctor.” Such distrust was often said to derive from an overwhelming motivation for profit by physicians, hospitals, and pharmaceutical companies.

Mistrust is also engendered by the perception of “flip-flopping” by medical experts. This perception was articulated in many ways, often using the example of changing advice during the COVID-19 pandemic about the necessity of wearing face masks. Study participants were aware that health authorities in the U.S. at first minimized the importance of face masks and then, as more data were accumulated about how the virus is spread, began strongly advocating for their use. A typical sentiment was, “They've changed their advice so many times that I've lost faith.” In addition, people often interpret such shifts in advice as signs of “corruption,” indicating that scientists are responding to political or industry pressures. In this vein, a participant explained that “I do not trust CDC [the Centers for Disease Control and Prevention] as they have become politically corrupted. Throughout the COVID-19 ordeal, CDC have continuously flip flopped, spewing out contradictory health advisories.”

Similarly, approval by the Food and Drug Administration (FDA) is neither persuasive nor reassuring for many vaccine hesitant Americans. Emergency Use Authorization (EUA) of the COVID vaccines was looked upon suspiciously, but even approval of a medication by FDA was not seen as providing much reassurance that a drug is safe. Participants think that the FDA is often wrong and that it has in the past approved medications that were later recalled. They cited thalidomide as an example, even though thalidomide was never approved by the FDA for use in the United States. This sentiment about the FDA was expressed by one participant who said, “the FDA can't be trusted blindly.” Another said “They [the FDA] approve bad things all the time.”

### Individualistic Reasoning

Participants generally focused on individual reactions to the vaccine and individual need, seeing vaccines as something only those at high risk of serious COVID-19 complications need to get. They did not typically speak about getting vaccinated as playing a part in a larger campaign or cause. Participants generally perceived risks as limited to short term, personal vaccine reactions, rather than taking into account the ongoing risk of a large pool of unvaccinated people and the ongoing risk of exposure to COVID-19. A very common form of vaccine hesitancy can be characterized as “wait-and-see,” the idea that a person will wait until many others have been vaccinated in order to assess if vaccines have any unforeseen dangers. One participant wrote, “My thoughts on the vaccine are that I will let it roll out for a while before I consider taking it. I would rather wait and see how the rest of the world reacts to it before I take it.”

Asian American participants were a notable exception to this, expressing greater interest in protecting the community by being vaccinated against COVID-19 than participants from other ethnic and racial groups.

### Vaccine Hesitancy Exists Along a Broad Continuum

**Figure 1:**
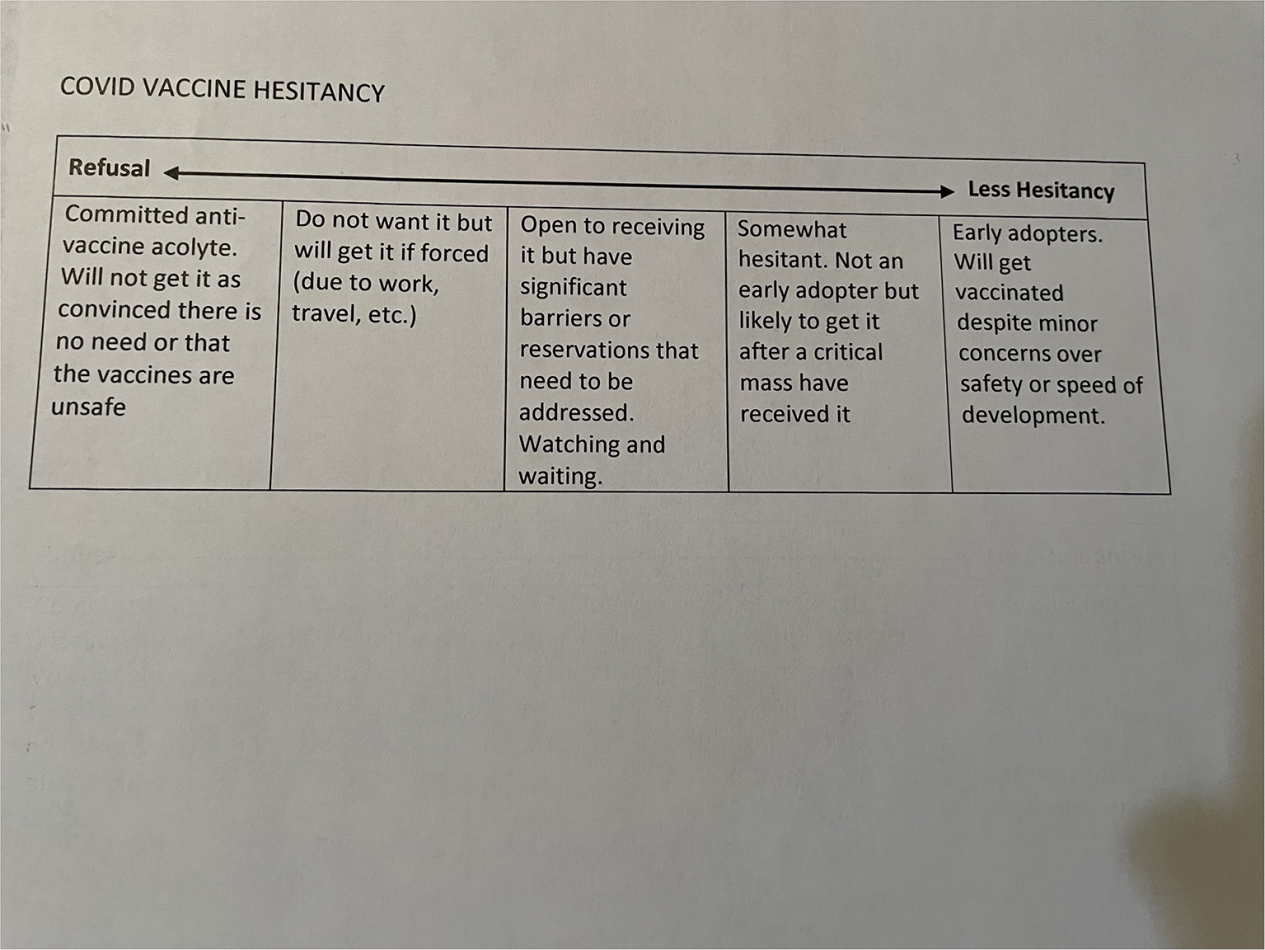
COVID vaccination hesitancy.

There is a continuum of vaccine hesitancy that ranges from fixed opposition to mild concern (see Figure 1). Although participants who expressed absolute vaccine refusal were excluded from this study, some nevertheless expressed a high degree of opposition. Much of this continuum comprises people who would ultimately like to get a COVID vaccine and are simply seeking some additional reassurance of its safety. As with the diffusion of any new technology, there is a substantial percentage of this group who can be categorized as “late adopters”—who do not like to see themselves as “guinea pigs” and embrace new technologies only *after* the majority of the population or the majority of their peer or reference group has already done so. For many, the ever-growing number of people being vaccinated without incident will likely serve as sufficient reassurance. For some, reassurance requires people they *know* safely getting the vaccine, or people in their particular communities or racial/ethnic groups being safely vaccinated. Many of these vaccine hesitant people may thus tip from hesitant to willing as vaccination numbers grow—especially among their peers. However, the research also suggests the power of additional, even unsubstantiated stories of adverse vaccine reactions to reinforce hesitancy in many people.

## Discussion

The bulletin board discussions about resistance to COVID-19 vaccines described here reinforced some things already known about vaccine hesitancy and revealed some important aspects about how vaccine hesitancy fits into a larger socio-ecological framework. We discuss here the implications of the six main themes that emerged (Table 2).

**Table 2:** Implications of COVID-19 Vaccine Hesitance.

**Research finding**	**Implication**
Personal physician most trust source of information	Utilize primary healthcare providers in public health communication efforts
Information comes from many sources, making people feel overwhelmed	Establish a national center for health communication
Negative stories are most “sticky”	Establish guidelines for journalists and editors on presenting stories that are not unnecessarily alarming
Mistrust of science and scientific institutions	Restore trust in federal, State, and local public health and scientific institutions
Individualistic reasoning	Develop effective messaging About community health
Continuum of vaccine hesitancy	Focus attention on those not yet fully committed; use Known role models; Counteract misinformation

Our research is consistent with findings that trust in primary care providers has been maintained despite the steady encroachment of commercialized medicine in the U.S. Given that physicians have less and less time to spend with their patients, it is somewhat surprising that people still find them to be their most trusted source of health information, yet survey studies (25, 26) and our online interviews show that to be the case. It is not clear that either healthcare policymakers or primary care physicians themselves fully recognize the potential influence doctors have to shape their patients' behaviors. This finding implies that more work should be done understanding how primary care physicians and other healthcare providers can play a more prominent role in delivering messages of public health importance, like the need to have a COVID-19 vaccine, to their patients. Such an expanded role would face the obstacle of the increasingly limited time physicians have to spend with patients and the fact that there is little physician reimbursement currently available for such tasks.

We found that information comes “at” people from every direction, leaving them feeling overwhelmed and confused. Unlike Canada or much of Western Europe, the United States does not have a strong, highly-trusted, publicly-supported news network. Instead, the private sector offers many choices, but also focuses more on entertainment and attention than informing. Our bulletin board results support the view that while a vast array of information sources may offer expanded opportunities to obtain information, these information sources also leave people feeling confused and overwhelmed. One solution to this might the creation of a nationally-recognized online science information center, which could provide people with reliable information and therefore reduce the relative proportion and influence of low-quality news sources. Ensuring access and exposure to high-quality sources of existing and newly emerging health information can help improve the overall health information environment.

Although there is a tendency to believe that storytelling, or presenting scientific consensus in the form of narratives, is lacks scientific rigor is therefore superficial, there is considerable evidence that stories are more persuasive in shaping attitudes and behaviors about health than are recitations of facts and data (27, 28). It is clear from our interviews that alarming stories are more “sticky” than data and that even a single story about a negative event, like an adverse side effect to a COVID-19 vaccine, is capable of forming an indelible memory trace that facts and data cannot easily dislodge. When conveying science to the general public, therefore, it may be important to consider a combination of both data and stories. For example, the story of a grandparent finally being able to hug her grandchild after getting a COVID-19 vaccine might have more salience in persuading people to be vaccinated than the nearly daily posting of the number of people who have died from COVID-19 or data on vaccine efficacy.

Participants in these interviews frequently accused public health agencies, like the CDC of “flip-flopping” and often used the changing advice on washing down surfaces and wearing face masks as examples. These changes in advice created the impression that the agencies are merely guessing or, even worse, deliberately attempting to manipulate the public without any basis in actual science. In fact, CDC guidance relied on emerging science as it became clear over the course of the first year of the pandemic that the virus that causes COVID-19 is spread by aerosolized particles. Nevertheless, the concept that practical health guidelines will inevitably change as scientific consensus changes with the emergence of new evidence seems not well understood.

The idea that once they are established, scientific theories and health guidelines should be fixed in place may be in part a function of the emphasis put on memorizing scientific facts during science education and on the tendency of the media to emphasize “breakthrough” findings rather than the grinding progress that science actually makes. These factors give the impression that once scientists reach a conclusion about something it must be permanently indisputable in order for science to maintain credibility. Science communications to the public need to convey an understanding that science does reach important conclusions, but that these are always subject to revision, especially when new technologies enable previously impossible insights to be realized.

Formulating guidelines to help journalists report on medical topics may be helpful to explain to the public how science works. Romer and Jamieson (29) have noted that headlines tend to exaggerate things that actually present relatively small risks and news stories often do not put these risks into context until several paragraphs of alarming narratives have been presented. For example, a headline that reads “Vaccine associated with risk of rare blood clot syndrome” is very different from a more accurate one that reads “Vaccines rarely associated with blood clots,” yet headlines tend to more often resemble the former example. Then, it may be several paragraphs before a reader is alerted to the fact that compared to the total number of vaccines received, an adverse event like blood clots is very rare. Although headlines and news stories are created to attract readers, it may still be possible to help journalists refrain from unnecessarily alarming the public about health risks by offering guidelines for the accurate reporting of health-related stories.

Because of the urgency of getting the COVID-19 pandemic under control, the FDA granted emergency use authorization (EUA) to vaccinations against SARS-CoV-2, rather than requiring the pharmaceutical companies to first apply for formal new drug approval. We found that neither method of authorization/approval carries much weight with our participants, who expressed skepticism about the FDA process and believe it often approves medications that turn out to be unsafe. In fact, vaccines that have been approved by the FDA have proven very effective and safe (30). There is a clear need for federal pharmaceutical regulatory authorities to educate the public about the process and procedures used to evaluate new medications, perhaps coupled with efforts to rebuild credibility in scientific institutions like the FDA and CDC that historically were well trusted by the public.

The “wait and see” attitude that our participants expressed about getting COVID-19 vaccines begs the question “wait for what?” When probed, those who maintained this individualistic attitude expressed the idea that they would have the vaccine only after many others are vaccinated without serious adverse consequences. The fact that the COVID-19 vaccines were each tested in tens of thousands of people before the FDA granted emergency use authorization for them seemed not to matter, but exactly how many successful vaccinations would be required to convince people they are safe was unclear. That is, participants often seemed to have an unreasonable expectation for what is required to reassure them a new drug or vaccine is safe. Even though millions of people have now received COVID-19 vaccines and serious adverse side effects are very rare, the focus of attention among our participants was often on just these rare adverse side effects instead of the overall safety record.

Interestingly, there was only one area in which we saw clear differences among ethnic groups: Asian Americans were more likely to adopt a view that being vaccinated was important to protect the community's health. All other groups demonstrated individualistic reasoning—other people should have the vaccine first in order to prove to the individual it is safe. One remedy for this could be messaging that stresses community health and the ability of individuals to protect the communities in which they live. Such an approach obviously requires experimental validation to see if it is likely to be effective.

Our final observation is that vaccine hesitancy exists along a continuum from those who are very strongly opposed to vaccination to those who seem poised to be vaccinated if they receive more reassurance about safety and efficacy. This continuum suggests that attention to increase vaccine uptake should be focused on providing reassurance and facts to the majority of vaccine-hesitant people who are still persuadable. An approach like this has been suggested that includes surveillance and typology of vaccine misinformation and intervention where that misinformation is posted (31).

We thus found that mistrust of COVID-19 vaccines is a phenomenon that exists within broader problems of mistrust of health institutions in general; that participants feel they face an overwhelming amount of information about health and science, much of which is incorrect or even manipulative; and that there is considerable misunderstanding about how science works. Many of our vaccine-hesitant participants had misconceptions about the way vaccines are developed, were prone to focus only on narratives of negative outcomes rather than on data, and were prone to individualistic reasoning while still believing themselves to be well informed about the vaccines. We have suggested several potential remedies for the broader issues that our results suggest, including:

1. Creating an online center for scientific information to provide unbiased and trustworthy information to the public;
2. Utilizing primary healthcare providers to convey public health messages; and
3. Establishing guidelines for journalists that reduce the tendency to provide narratives that focus only on rare negative outcomes.

A limitation of our study is that the participants do not comprise a representative sample and were selected from three specific regions in the U.S. While this research can provide insight into the range of opinions that exist among the population of vaccine-hesitant people in the U.S., it cannot address the frequency with which any particular opinion about vaccinations or any given category of hesitancy exists within the general population. However, to the extent that the information gleaned from the participants in this study reveals important problems with how attitudes and behaviors about public health and vaccinations are formed and maintained, our findings and recommendations may be worthy of consideration.

## Data Availability Statement

The raw data supporting the conclusions of this article will be made available by the authors, without undue reservation.

## Ethics Statement

The studies involving human participants were reviewed and approved by 1. Ethical an Independent Review Services; 2. Weil Cornell College of Medicine. The patients/participants provided their written informed consent to participate in this study.

## Author Contributions

DS, JG, and SG: conceived and planned study. JG: wrote initial draft of manuscript. WS and NG: supervised data collection. DS and JG: obtained funding. All authors reviewed and approved manuscript.

## Funding

Funding for this project was provided by the Robert Wood Johnson Foundation, Grant ID # 76935. The funder provided advice in the broad planning of this project and open access publication fees.

## Conflict of Interest

WS and NG are employed by company Fluent LLC. The remaining authors declare that the research was conducted in the absence of any commercial or financial relationships that could be construed as a potential conflict of interest.

## Publisher's Note

All claims expressed in this article are solely those of the authors and do not necessarily represent those of their affiliated organizations, or those of the publisher, the editors and the reviewers. Any product that may be evaluated in this article, or claim that may be made by its manufacturer, is not guaranteed or endorsed by the publisher.
