# Corrigendum: Implications of COVID-19 Vaccine Hesitancy: Results of Online Bulletin Board Interviews

**DOI:** 10.3389/fpubh.2022.868438

**Published:** 2022-03-08

**Authors:** Jack M. Gorman, Sara E. Gorman, William Sandy, Nellie Gregorian, David A. Scales

**Affiliations:** ^1^Critica Inc., Bronx, NY, United States; ^2^Fluent LLC, New York City, NY, United States; ^3^Department of Medicine, Weil Cornell Medical College, New York City, NY, United States

**Keywords:** COVID-19, vaccine, online, hesitancy, trust

In the original article, we neglected to include the funder the Cornell Center for Social Science, which provided support to change the focus groups to online bulletin boards after the onset of the COVID-19 pandemic.

In the published article, there was an error regarding the affiliations for Jack M. Gorman, Sara E. Gorman, William Sandy, Nellie Gregorian and David Scales. While the first four authors were incorrectly listed as affiliated with Weill Cornell, the last author should also have Critica as an affiliation.

The authors apologize for these errors and state that they do not change the scientific conclusions of the article in any way. The original article has been updated.

## Publisher's Note

All claims expressed in this article are solely those of the authors and do not necessarily represent those of their affiliated organizations, or those of the publisher, the editors and the reviewers. Any product that may be evaluated in this article, or claim that may be made by its manufacturer, is not guaranteed or endorsed by the publisher.

